# Cardiac CT Improves Outcomes in Stable Coronary Heart Disease: Results of Recent Clinical Trials

**DOI:** 10.1007/s12410-017-9411-7

**Published:** 2017-03-25

**Authors:** Michelle C. Williams, Alastair Moss, Edward Nicol, David E. Newby

**Affiliations:** 1 University of Edinburgh/British Heart Foundation Centre for Cardiovascular Science, Chancellor’s Building, 49 Little France Crescent, Edinburgh, EH164SB UK; 2 Royal Brompton and Harefield NHS Trust Departments of Cardiology and Radiology, London, UK

**Keywords:** Computed tomography, Chest pain, Angina, Coronary heart disease

## Abstract

**Purpose of Review:**

The purpose of this study was to review the recent randomised controlled trials of coronary computed tomography angiography (CCTA) for patients with stable coronary artery disease.

**Recent Findings:**

The initial results and subsequent papers from the SCOT-HEART (Scottish COmputed Tomography of the HEART) and PROMISE (PROspective Multicentre Imaging Study for Evaluation of chest pain) trials have shown that CCTA is a safe and appropriate addition to standard care or alternative to functional testing. The SCOT-HEART study showed that CCTA changes diagnoses, improves diagnostic certainty, changes management, leads to more appropriate use of invasive coronary angiography, and reduces fatal and non-fatal myocardial infarction. A meta-analysis of the four randomised controlled trials showed that CCTA leads to a major reduction in myocardial infarction in patients with stable chest pain.

**Summary:**

CCTA is now an established technique for the assessment of coronary artery disease. Recent ‘test and treat’ randomised controlled trials have shown that CCTA guided changes in management can improve clinical outcomes.

## Introduction

Coronary computed tomography angiography (CCTA) is now an established technique for the assessment of patients with suspected coronary artery disease (CAD). It has an excellent diagnostic accuracy for the identification of CAD, with a high negative predictive value [1]. Large-scale registry studies have shown the prognostic utility of CCTA in the identification of both obstructive disease and non-obstructive atherosclerotic plaque. However, recent clinical studies have moved beyond the assessment of diagnostic accuracy or registry studies. This review discusses the outcome-based research that has established the role of CCTA in the assessment of patients with suspected coronary heart disease.

A normal CCTA is associated with a good prognosis in large registry studies of patients with suspected CAD [2–4]. Both the presence and the extent of obstructive CAD were associated with a worsening prognosis in the COronary CT Angiography EvaluatioN For Clinical Outcomes: An InteRnational Multicenter (CONFIRM) registry [5]. In addition, the extent of non-obstructive coronary artery atherosclerotic plaque was a predictor of mortality in several registry studies [4, 6, 7] and the use of statin therapy at baseline was associated with reduced mortality for patients with non-obstructive CAD in the CONFIRM study [6]. The lack of randomisation in registry studies means that there is major confounding by indication, and that they cannot determine the clinical utility of CCTA or the effect of treatment decisions based on CCTA results. More recently, several large ‘test and treat’ randomised controlled trials have been performed to address these issues, including the PROMISE and SCOT-HEART studies.

### PROMISE

The PROMISE (PROspective Multicentre Imaging Study for Evaluation of chest pain) trial recruited 10,003 symptomatic stable outpatients who were due to undergo non-invasive investigation for suspected CAD [8••]. Participants were randomised to undergo either anatomical assessment with CCTA or functional testing with exercise electrocardiography, stress echocardiography or radionucleotide perfusion imaging (Table 1).

**Table 1:** Study design of the four randomised studies of CCTA

	PROMISE	SCOT-HEART	CAPP	Min et al.
Recruiting centres	193	12	1	1
Study groups	CCTA vs functional testing (ETT, stress echo or radionucleotide perfusion imaging)	CCTA and standard care vs standard care alone	CCTA vs ETT	CCTA vs MPS
Primary endpoint	Composite of all-cause mortality, myocardial infarction, hospitalisation for unstable angina, and major complications of cardiovascular procedures or diagnostic testing	Proportion of patients diagnosed with angina pectoris secondary to coronary heart disease at 6 weeks	Difference in symptoms from baseline to 3 months	Angina-specific health status
Follow-up duration (months)	25	20	12	55 days

*ETT* exercise electrocardiography, *echo* echocardiography, *MPS* myocardial perfusion single photon emission

The mean age of the participants was 61 years, 53% were female and the pre-test probability of obstructive CAD was 53%. Over one quarter of patients (27%) presented with a primary symptom other than chest pain, such as breathlessness or fatigue. The characterisation of chest pain was typical angina for 12%, atypical angina for 78% and non-anginal chest pain for 11% (Table 2).

**Table 2:** Demographic details of patients recruited into the four randomised studies of CCTA

		PROMISE	SCOT-HEART	CAPP	Min et al.
Number		10,003	4146	448	180
Age (years)		61	57	59	56, 59
Female (%)		53	44	45	44
Pre-test probability (%)		53	47	45, 48	–
Chest pain (%)	Typical angina	12	35	34	32
	Atypical angina	78	24	8	23
	Non-anginal chest pain	11	41	67	27

The primary outcome of the PROMISE study was a composite of all-cause mortality, myocardial infarction, hospitalisation for unstable angina and major complications of cardiovascular procedures or diagnostic testing. At 12 months of follow-up, the risk of death or non-fatal myocardial infarction was lower in the CCTA group than in the functional imaging group (hazard ratio (HR) 0.66, 95% confidence interval (CI) 0.44–1.00, *P* = 0.049). However, at 25 months of follow-up, there was no difference in the primary outcome between the two groups (events 3.3 vs 3.0%, HR 1.04, 95% CI 0.83–1.29, *P* = 0.75). Thus, PROMISE showed that CCTA is a safe alternative to functional testing in a low-risk population with similar outcomes in both groups after 2 years of follow-up.

### SCOT-HEART

The SCOT-HEART (Scottish COmputed Tomography of the HEART) trial randomised 4146 outpatients with suspected angina due to CAD to standard care or standard care plus CCTA [9]. Participants were recruited from cardiology outpatient rapid access chest pain clinics (RACPC) and 47% of all eligible patients were recruited, including patients with atrial fibrillation, high calcium score and high body mass index [10••].

The mean age of participants was 57 years, 44% were female and the pre-test probability of obstructive coronary heart disease was 47%. The presence of typical angina was higher in the SCOT-HEART trial as compared to the PROMISE trial (35 vs 12% of participants). Atypical angina was the presenting complaint in 24% of patients (cf. 78% in PROMISE) and 41% (cf. 11%) had non-anginal chest pain (Table 2).

The primary endpoint of the SCOT-HEART trial was the certainty of the diagnosis angina pectoris secondary to significant CAD at 6 weeks (Table 1). At 6 weeks, the diagnosis was changed in 23% of patients undergoing CCTA compared to 1% in the standard care group (*p* < 0.0001). CCTA improved the certainty of the diagnosis for both the presence of CAD and the diagnosis of angina due to (CAD) (RR 2.56, 95% CI 2.33–2.79, *P* < 0.0001 and RR 1.79, 95% CI 1.79, 1.62–1.96, *P* < 0.001). CCTA increased the frequency of the diagnosis of CAD (relative risk (RR) 1.09, 95% CI 1.02–1.17, *P* = 0.0172), but tended to decrease the frequency of the diagnosis of angina due to significant CAD (RR 0.93, 95% CI 0.85–1.02, *P* = 0.1289).

At 1.7 years of follow-up, the CCTA group had a 38% lower rate of fatal and non-fatal myocardial infarction as compared to the control group, but this difference did not quite reach statistical significance (26 vs 42, HR 0.62, 95% CI 0.38–1.01, *P* = 0.0527) [10••]. The overall event rate was low, similar to the PROMISE study, occurring in just 2% of participants. However, in a post hoc landmark analysis censored to the median time of treatment alteration (50 days), there was a 50% reduction in fatal and non-fatal myocardial infarction in the CCTA group (17 vs 34, HR 0.50, 95% CI 0.28–0.88, *P* = 0.020) [11••].

### Other Studies and Meta-analysis

There are two other smaller randomised controlled trials which have recently assessed CCTA in patients with stable chest pain: the CAPP trial [12•] and a study by Min et al. [13•].

The CAPP trial randomised 500 patients with stable chest pain who were referred to a RACPC for assessment [12•]. Patients were randomised to either undergo exercise stress electrocardiogram (ETT) or CCTA (Table 1). The mean age was 59 years, 45% were female, and the pre-test probability of significant CAD was 45% in the ETT group and 48% in the CCTA group (*P* = 0.34) (Table 2). The primary endpoint was the difference in symptoms from baseline to 3 months between the two groups, assessed using the Seattle Angina Questionnaire. They identified no difference in major adverse cardiac events between the ETT and CCTA groups.

The study by Min et al. randomised 180 patients with stable chest pain from cardiology outpatient clinics to undergo CCTA or myocardial perfusion single photon emission CT (MPS). [13•] (Table 1) The mean age was 56 years for the CCTA group and 59 years for the MPS group (*P* = 0.04), and 44% were female (Table 2). Pre-test probability was not provided but 32% had typical angina, 23% atypical angina and 27% non-anginal chest pain. The primary endpoint was the “angina-specific health status”. No patients had a myocardial infarction or died in either group during the follow-up of 55 days.

A meta-analysis has combined the primary results of PROMISE, SCOT-HEART, CAPP and the study by Min et al. [14•]. It identified that compared to standard care, the use of CCTA was associated with a reduction in the annual rate of myocardial infarction (rate ratio 0.69, 95% CI 0.49–0.98, *P* = 0.038), but there was no difference in all-cause mortality [14•]. This 31% relative risk reduction in myocardial infarction occurred with CCTA despite the relatively low rate of events in the individual studies over a median of 2 years of follow-up.

### Effect of CCTA on Downstream Investigations

In the SCOT-HEART trial, CCTA led to a change in the planned investigations in 15% of participants, compared to 1% in the standard care group (*P* < 0.0001) [10••]. For some participants, planned investigations were cancelled, whilst for others, new investigations were organised, primarily invasive coronary angiography (ICA). Overall, the rates of ICA were similar between groups in the SCOT-HEART study (409 vs 401, *P* = 0.451). In the study by Min et al., there were also similar rates of ICA between the CCTA and the MPS groups [13•]. However, in the PROMISE study, there was an increase in the number of patients undergoing ICA in the CCTA group compared to the functional imaging group (12.2 vs 8.1% at 90 days). In both the PROMISE and SCOT-HEART, there was a reduction in the proportion of patients with normal ICA [8••, 11••]. In SCOT-HEART, normal or non-obstructive CAD was identified at invasive coronary angiography in 29% of patients in the CT group compared to 41% of patients in the standard care group. Normal coronary arteries at invasive coronary angiography were identified in SCOT-HEART in 6.6% of patients in the CT group compared to 19.6% in the standard care group. In PROMISE, normal or non-obstructive CAD was identified at invasive coronary angiography in 28% of patients in the CT group compared to 53% in the control group. This shows that CCTA can be used to select appropriately patients for ICA and can reduce the number performed in patients with normal coronary arteries.

### Medical Treatment

In the SCOT-HEART study, treatment was changed in 23% of patients in the CCTA group compared to 5% in the standard care group (*P* < 0.0001). This included an increase in the use of preventative therapies (such as statins, Aspirin and ACE inhibitors) and decrease in the use of anti-anginal therapies [10••, 11••]. Min et al. also demonstrated that patients in the CCTA arm had a subsequent increased use of aspirin and statins [13•]. CCTA can identify non-obstructive CAD which other investigations may not identify. It is therefore not surprising, nor a new finding, that CCTA is associated with an increased use of preventative medications in such patients [15]. However, a major strength of CCTA is its negative predictive value and therefore the cessation of unnecessary medications in patients with normal coronary arteries is also an important outcome after CCTA.

### Revascularisation

In the PROMISE trial, there was an increase in the numbers of patients going on to have revascularisation after invasive coronary angiography in the CCTA group (6.2 vs 3.2%, *P* < 0.001) [8••]. In the SCOT-HEART trial, there was also a statistically non-significant trend towards an increased proportion of revascularisation in the CCTA group (11.2 vs 9.7%, *P* = 0.0611) [10••]. Therefore, CCTA can be used to more appropriately select patients who require further invasive investigation and treatment.

### Cost Analysis

In both the SCOT-HEART and the PROMISE trials, there was a small increase in cost in the CCTA group. In the PROMISE study, the mean cost difference at 90 days was $254 (95% confidence interval—$634 to $906) [16], and in the SCOT-HEART study, the difference was $462 (95% confidence interval $303 to $621) at 6 months [11••]. In the PROMISE study the cost difference was due to the increased use of ICA and revascularisation in the CCTA group. However, in the SCOT-HEART study the difference was attributed to the cost of CCTA itself, as the rate of ICA was not statistically different between groups [11••]. The cost of CCTA technology continues to fall and therefore this small difference in cost between investigation strategies is likely to continue to decrease in the future.

### Symptoms

In SCOT-HEART, PROMISE and CAPP trials, symptoms improved in both groups of patients indicating general satisfaction after attendance at the RACPC or cardiology outpatient clinic [10••, 12, 17]. In the SCOT-HEART trial, there was no difference in symptoms at 6 week between CCTA and standard care groups [10••]. In the PROMISE trial, there was no difference in symptoms over 2 years of follow-up between the CCTA group or functional imaging group [17]. In the study by Min et al., there was no difference in angina symptoms between groups at 55 days [13•]. However, in the CAPP study, there was a larger improvement in angina symptoms in the CCTA group compared to the ETT group at 3 and 12 months [12•]. This suggests that there may be improved management and patient satisfaction after CCTA as compared to ETT. Whilst CCTA undoubtedly improves diagnosis and allows the optimisation of treatment for patients, CCTA findings can also provide potential uncertainty and anxiety, particularly for those patients who are subsequently recommended to start life-long preventative treatments for non-obstructive CAD. Therefore, it is important to consider potential patient anxiety when communicating the results of the CCTA.

### Radiation Dose

**Fig. 1:**
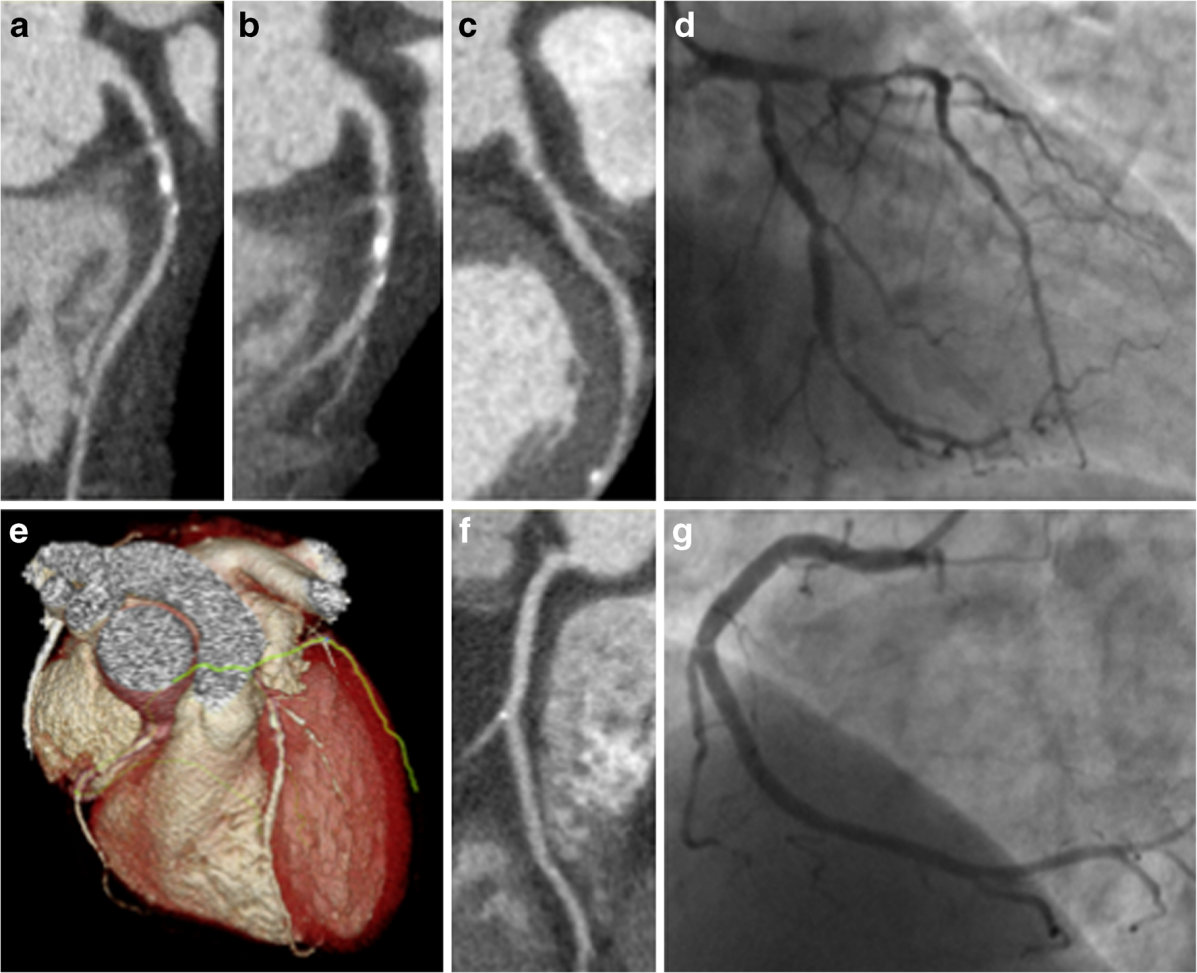
An example of low radiation dose CCTA with comparison to invasive coronary angiography from the SCOT-HEART study. This 60-year-old male patient with no previous history of cardiovascular disease and no cardiovascular risk factors presented with chest pain. CCTA identified severe disease in the left anterior descending coronary artery (**a**), first diagonal (**b**) and circumflex artery (**c**) which was confirmed on invasive coronary angiograph (**d**). Mild non-obstructive disease was identified in the right coronary artery (**f**, **g**). **e** Shows a three-dimensional reconstruction of the heart from the CT. The patient underwent revascularisation. The total dose length product for CCTA and calcium scoring CT was 358 mGy cm (5 mSv using the 0.014 mSv/mGy cm conversion factor)

In the PROMISE trial, the overall radiation exposure was higher in the CCTA group (mean 12 mSv (milliseverts) vs 10.1 mSv, *P* < 0.001). However, as 33% of patients in the functional testing group did not have any radiation exposure at all, and therefore, the median cumulative radiation exposure per patient was lower in the CCTA group (10.0 vs 11 mSv). In the SCOT-HEART trial, the median radiation dose for CCTA and non-contrast coronary artery calcium score was 4.1 (interquartile range 3.0–5.6) mSv. More than one third of the radiation exposure in the SCOT-HEART trial was attributed to the coronary artery calcium score. The additional benefit of performing a non-contrast scan for calcium scoring in patients who are undergoing CCTA is low, and indeed, in many cases, this additional examination is not required. The mean radiation dose in the CCTA group of the CAPP trial was 5.37 mSv. In the study by Min et al.*,* the CCTA group had a significantly lower radiation dose than the MPS group (7.4 vs 13.3 mSv, *P* < 0.001). CCTA can now be performed at a low radiation dose for patients with a range of heart rates and body size (Fig. 1).

### Plaque Characterisation

In addition to assessing stenosis severity, CCTA can assess the constituents and morphology of atherosclerotic plaques. Motoyama et al. identified that the presence of low attenuation plaque, positive remodelling and spotty calcification were associated with an increased risk of acute coronary syndrome [18]. A meta-analysis of subsequent studies found a significantly higher risk of acute coronary syndromes in patients who had high-risk plaque (odds ratio (OR) 12.1, 95% CI 5.24–28.1, *P* = 0.0001) [19]. However, the use of these plaque markers in randomised outcome-based research studies have not yet been assessed.

### Functional Assessment

Cardiac CT can assess myocardial perfusion using static or dynamic techniques with good diagnostic accuracy compared to other imaging modalities [20]. Computational fluid dynamic models can estimate pressure and flow within the coronary arteries. These estimates can be used to calculate non-invasive fractional flow reserve (FFR). The use of non-invasive FFR based on CCTA images may reduce the rate of normal invasive coronary angiograms, lower costs reduce radiation exposure, improved quality of life and improve the detection of lesions causing ischaemia [21–24].

## Conclusion

CCTA is a safe diagnostic test that can be performed at a low radiation dose and is now widely available. Recent clinical trials have established the role of CCTA in the assessment of patients with suspected CAD. The use of CCTA not only changes the diagnosis but it also alters management that ultimately reduces the future risk of fatal and non-fatal myocardial infarction. National guidelines, such as the UK NICE guidelines, have recently changed to incorporate CCTA as the first-line test in stable chest pain (discussed by Moss et al. in another paper in this issue) and other international guidelines are likely to follow suit in time.

## Abbreviations

CCTA: Coronary computed tomography angiography
CI: Confidence interval
ETT: Exercise tollerance test
HR: Hazard ratio
ICA: Invasive coronary angiography
mSv: Millisievert
MPS: Myocardial perfusion single photon emission
OR: Odds ratio
RACPC: Rapid access chest pain clinic
RR: Relative risk
